# Hospitalizations After Bicycle Accidents: Injury Patterns, Severity and Costs

**DOI:** 10.3390/ijerph23060788

**Published:** 2026-06-11

**Authors:** José Antonio Guerrero Serrano, Samuel Lozano Martín, Julia Sánchez García, Marta Arroyo Hernández, Pedro Caba Doussoux

**Affiliations:** 1 Department of Orthopaedic Surgery, Hospital Universitario 12 de Octubre, 28041 Madrid, Spain; martaarroyohdz@gmail.com (M.A.H.); pedrocabado@gmail.com (P.C.D.); 2 Faculty of Medicine, Universidad Complutense de Madrid, 28040 Madrid, Spain; samueloz@ucm.es (S.L.M.); juliasangar2@gmail.com (J.S.G.)

**Keywords:** bicycle, cycling accident, hospitalization, injury severity, traumatic brain injury, helmet, costs

## Abstract

**Highlights:**

**Public health relevance—How does this work relate to a public health issue?**

- The widespread increase in environmental awareness, the growing recognition of the health benefits of cycling, and the emergence of new mobility trends have contributed to the more frequent use of bicycles for both recreational and commuting purposes.
- The rise in bicycle use has been paralleled by an increase in the number of cycling accidents.

**Public health significance—Why is this work of significance to public health?**

- The increasing number of hospitalizations due to cycling accidents represents a potential trauma burden on the healthcare system, highlighting the need for a better understanding of epidemiology, severity, and costs associated with cycling injuries.
- The association between injury severity and increased hospital costs highlights the need to raise awareness of the economic burden of cycling-related trauma on the healthcare system.

**Public health implications—What are the key implications or messages for practitioners, policy makers and/or researchers in public health?**

- Helmet advocacy and promotion of helmet use are warranted, given their association with less severe neurological injuries.
- Preventive strategies should target collision reduction through road safety policies and user awareness, as collisions are typically associated with injuries of greater severity.

**Abstract:**

Background Although cycling has definite health benefits, it is certainly not a risk-free activity; its increasing use is associated with a rise in accidents. This study aims to characterize cycling injuries and their associated factors in a tertiary trauma center, including injury severity, accident circumstances, and in-hospital costs. Methods: A retrospective observational study was conducted on patients over 15 years of age hospitalized after a cycling accident. Collected variables included the characteristics of the accident, the epidemiology of musculoskeletal injuries, helmet use, injury severity as assessed using the Abbreviated Injury Scale (AIS), the Injury Severity Score (ISS), and costs. Results: A total of 131 patients were included, of whom 90.8% were male, with a mean age of 43.2 ± 14.1 years. Most accidents were due to falls (83.7%). Accidents occurred in urban areas (56.3%), inter-urban roads (28.1%), and rural areas (15.6%). Upper limb fractures, particularly clavicle fractures (13.7%), were the most frequent injuries (31.0%). Traumatic brain injury (TBI) was present in 30.0% of patients, and 17.6% were polytraumatized. Injury severity was higher in males (*p* = 0.009) and in collisions compared with falls (*p* = 0.033). It was also correlated with length of hospital stay (r = 0.376). Patients with TBI exhibited significantly higher ISSs (*p* < 0.001). Helmet use was reported in 71.1% of patients and was more frequent in rural areas (*p* < 0.001) and associated with lower neurological AIS scores (*p* = 0.031). The mean cost per patient was €8545 ± 15,298, increasing with severity of injury (*p* < 0.001), and was higher in polytraumatized patients (*p* < 0.001) and in those with TBI. Conclusions: Cycling accidents most frequently resulted in upper limb fractures. Helmet use was more common where mandatory and was associated with less severe neurological injuries but not with a lower incidence of TBI. Costs increased with injury severity, particularly in patients with TBI and longer hospital stays.

## 1. Introduction

In line with many other countries, Spain has recently experienced a substantial increase in the use of the bicycle, particularly during the COVID-19 pandemic. This change in mobility patterns has contributed to environmental sustainability, a reduction in urban traffic congestion and an improvement in public health, including a reduction in cardiovascular and other disease-related risk factors [1,2,3].

Nonetheless, cycling is certainly not a risk-free activity. In fact, the increase in bicycle use has been accompanied by a higher accident rate. In 2022, Spain was the scene of a total of 8106 bicycle accidents, 3% more than those reported in 2019 [4,5,6]. Cyclists involved in these accidents presented with a wide range of injuries, typically of limited severity, occurring generally in the limbs [7,8,9,10]. However, such factors as speed, the dynamics of the accident and the potential involvement of motor vehicles could result in injuries requiring hospital admission [8]. Nearly 9% of cyclists involved in accidents in 2022 in Spain had to be hospitalized, a percentage above that recorded in 2019 [6]. The clinical, functional and quality-of-life-related impact suffered by these patients is typically compounded by a significant economic burden resulting from medical transportation, emergency care, medical treatments and, in some cases, hospitalization [11,12].

Of all the injuries sustained by cyclists, those affecting the head, and particularly traumatic brain injuries (TBIs), are the most feared on account of their severity and associated mortality rate [8,10]. Helmets are the most effective protection against such injuries. However, in spite of the fact that their use is compulsory on all inter-urban roads in Spain and that the government launches regular campaigns to encourage their use, a certain perception of helmet discomfort still persists, particularly in connection with the use of the device in urban areas [13,14]. This is exemplified by the fact that 30.4% of cyclists who died in 2024 were not wearing a helmet at the time of the accident [15].

The literature available on cycling accidents in Spain is scarce; most of it is limited to institutional reports that often group several types of road accidents under the same category [2,6,16]. The present retrospective observational study sought to characterize cycling injuries treated in a Spanish tertiary trauma center by describing the demographic profile of injured cyclists, the circumstances surrounding the accidents, and the epidemiology and severity of the musculoskeletal injuries sustained. Furthermore, the study aimed to analyze the relationship between injury severity and various cyclist- and accident-related factors. In addition, helmet use was evaluated as a key safety measure by assessing its association with the accident’s characteristics, the severity of injuries, and the patients’ in-hospital course. Finally, the study assessed in-hospital costs and their relationship with injury severity and clinical outcomes. These findings may provide useful clinical evidence to support public health authorities in the development of preventive strategies, the promotion of safety measures such as helmet use, and the optimization of healthcare resource planning.

## 2. Materials and Methods

### 2.1. Study Design

This is a retrospective observational study on a group of patients who required hospitalization following a cycling accident. The study was approved by the Ethics Committee of the Hospital Universitario 12 de Octubre (Madrid, Spain) (approval code: 26/217, date of approval: 14 April 2026). As no additional clinical interventions were planned, the Committee granted an informed consent waiver to ensure the feasibility of the study. This study was reported following the Strengthening the Reporting of Observational Studies in Epidemiology (STROBE) guidelines [17]. 

### 2.2. Data Sources

The data for the study were obtained from the Registro de Actividad de Atención Especializada (RAE-CMBD), an administrative hospital-based database managed by the Spanish Health System [18]. The specific documents retrieved were reports prepared by the 12 de Octubre Hospital upon admission of persons involved in cycling accidents (ICD-9-CM code E826.1). Additionally, severity scores were obtained from reviewing electronic medical records. As the information related to accidents (location, mechanism of injury, bicycle type and helmet use) is not usually collected systematically in medical records, given the urgency to provide appropriate treatment, telephone interviews were conducted between January and May of 2023 to obtain the missing information. The in-hospital economic costs were made available by the hospital’s accounting department.

### 2.3. Selection Criteria

All patients admitted to the Hospital Universitario 12 de Octubre following a cycling accident taking place in the Madrid region between 1 January 2015 and 31 December 2021 were included in the study. The exclusion criteria were injury-coding-assignment errors and patients under 15 years of age, since children with traumatic injuries are managed in our institution through a dedicated care pathway, whereas adolescents aged 15 years and above are treated according to adult trauma protocols. In cases where patients were unable to recall details of the accident during the telephone interview, the corresponding variables were not recorded and were treated as missing data in subsequent analyses.

### 2.4. Variables

A record was made of the patients’ demographic variables (sex and age) and the characteristics of the accident (date, location, mechanism of injury, bicycle type, and whether the cyclist was wearing a helmet or not). The following locations were considered: inter-urban roads, defined as paved and well-signposted thoroughfares connecting different population centers; rural areas, characterized by unpaved or unsignposted paths located away from population centers; and urban areas, which included paved surfaces located within population centers, such as roads, bike lanes or recreational areas. Mechanisms of injury were, in turn, classified into two categories: collision with an external agent, such as a vehicle, another cyclist, a pedestrian, or the road surface, and fall, defined as a loss of stability by a cyclist caused by reasons other than a collision (avoiding obstacles, avoiding a collision, presence of objects stuck in the bicycle wheels, loss of balance, or malfunctioning of the bicycle). Variables related to the patients’ hospitalization (type of transport used, department the patient was admitted to, length of hospital stay, and number of surgeries required), and to their musculoskeletal injuries were also collected. The severity of these injuries was evaluated using the Abbreviated Injury Scale (AIS) and the Injury Severity Score (ISS). The ISS was used to categorize patients into four severity groups: minor (0–8), moderate (9–15), severe (16–24) and very severe (≥25). Patients with an ISS score equal to or higher than 16 were classified as polytraumatized [19]. All medical costs, as well as the patients’ mortality and their cause of death, were also recorded.

### 2.5. Statistical Analysis

All statistical analyses were performed using R software v. 4.4.2 (R Development Core Team) [20]. Given the varying sample sizes across variables, analyses were performed using the available data, and percentages were calculated excluding missing values. Descriptive statistics were calculated for all variables, including the number of observations available for each of them (N) as well as the relevant measures of central tendency and dispersion.

Comparisons were carried out to determine whether accident frequencies varied according to their temporal distribution (day of the week, month, and year). Additional comparisons were performed to explore potential associations between cyclist, accident and in-hospital course-related variables, and the presence of TBI and polytrauma, with injury severity assessed by the ISS. Helmet use was analyzed in relation to temporal distribution, injury characteristics, presence of a head fracture, injury severity, and in-hospital treatment course to evaluate its potential association with injury patterns and clinical outcomes. Finally, in-hospital costs were analyzed according to injury severity indicators and clinical course variables, including polytrauma, TBI, neurological AIS score, ISS, helmet use, number of surgeries, and length of hospital stay, due to their potential influence on healthcare resource utilization.

Qualitative variables were analyzed by means of Pearson’s chi-squared test or Fisher’s Exact Test when expected cell frequencies were <5. Differences between mean values were studied using either Wilcoxon’s test or Student’s *t*-test, depending on the normality of the sample. In the case of variables with more than two categories, the Kruskal–Wallis test was used to determine the presence of statistically significant differences in the distribution of patients across groups. If significant differences were found, Wilcoxon’s signed rank test adjusted with Bonferroni correction was applied to control for Type I errors resulting from multiple comparisons.

Furthermore, the correlation of the ISS and costs with the continuous variables was analyzed using Pearson’s correlation coefficient, considering the following cut-off points: r ≤ 0.25 (weak), 0.25 < r ≤ 0.50 (moderate), and r ≥ 0.75 (strong) [21].

Statistical significance was set at a *p*-value < 0.05 in all cases.

## 3. Results

### 3.1. Cycling Accidents: Characteristics and Severity of the Injuries Sustained

**Figure 1:**
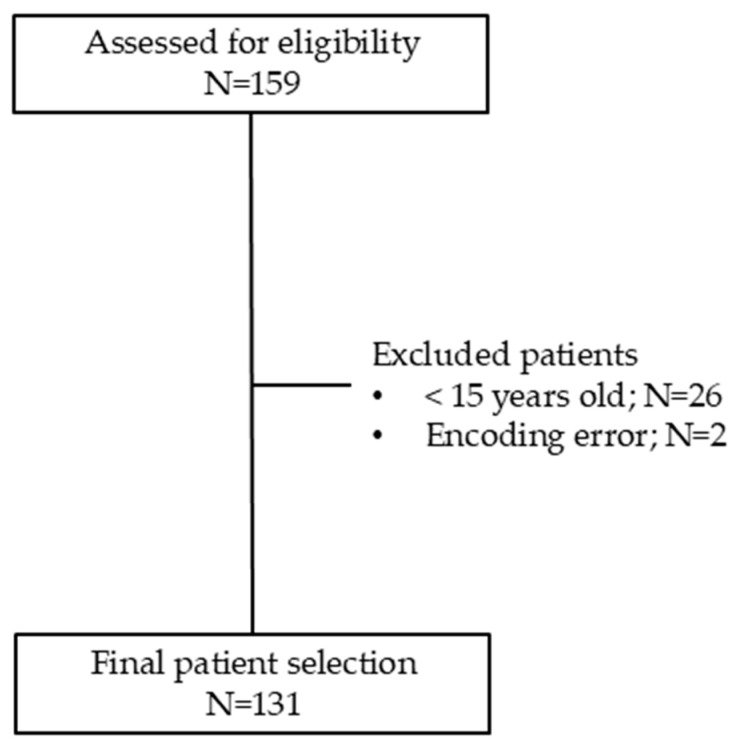
Patient selection STROBE flowchart.

A total of 159 cycling accidents were recorded during the study period, of which 26 were excluded because cyclists were below the stipulated age, and another two were excluded because of code-assignment errors (Figure 1). Of the remaining sample of 131 patients, 90.8% were male. Overall, the median age was 45 years (IQR: 32.5–53.0), with 48.9% of patients aged between 36 and 55 years.

**Figure 2:**
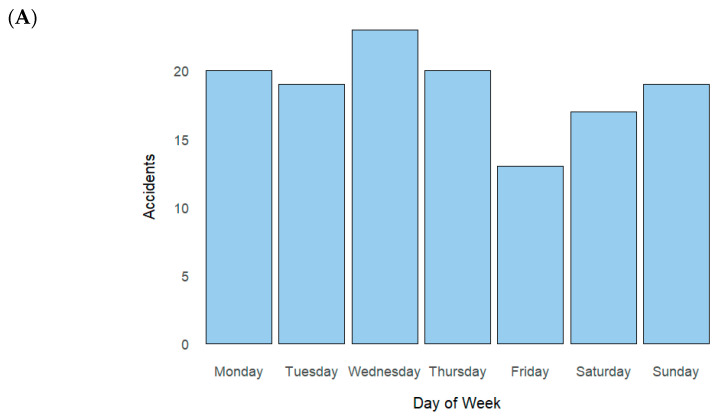

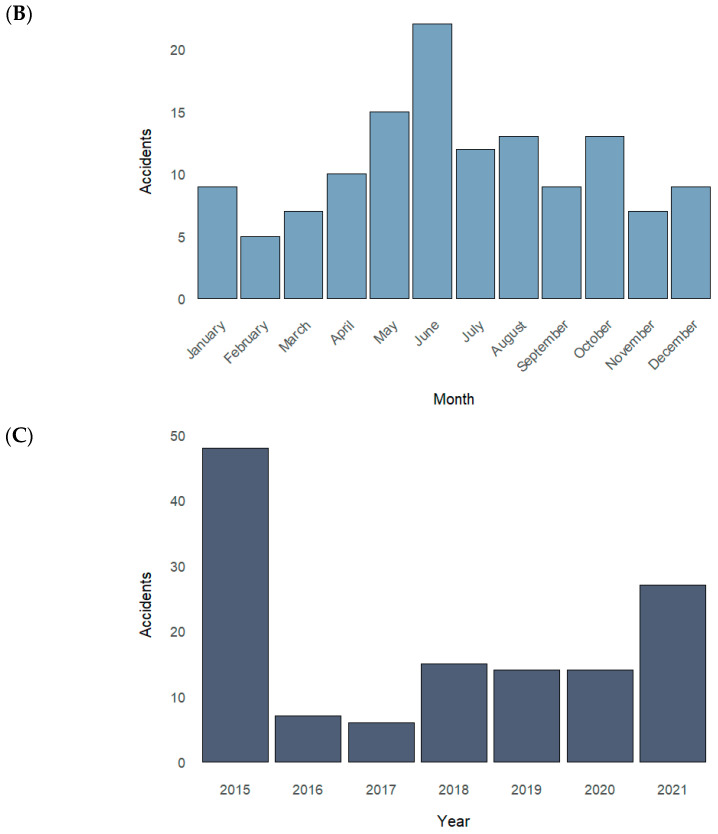
Distribution of accidents across the days of the week (**A**), the months of the year (**B**) and the years considered for the analysis (**C**).

The distribution of accidents over the study period is shown in Figure 2. While no statistically significant differences were found in the cycling accident frequencies between the different days of the week (*p* = 0.167) or the different months in the year (*p* = 0.256), such differences were observed between the years under analysis (*p* < 0.001).

Variables related to the characteristics of the cycling accident and the patients’ in-hospital course are presented in Table 1. Thirty-three percent of the patient population was made up of males ≥ 50 years who suffered an accident in rural or inter-urban areas.

Musculoskeletal injuries, as well as the main severity-related parameters, are shown in Table 2. A total of 217 injuries were recorded, of which 184 were fractures, 31 in the lower limbs (16.8%), 13 in the spine (7.1%), 57 in the upper limbs (31.0%), 24 in the torso (13.0%), and 59 (32.1%) in the head. In addition, eight fracture-dislocations and seven dislocations were observed, and 39 patients (29.8%) presented with a TBI. Although over half of the patients presented with low-severity injuries, 64.9% had to undergo at least one surgery. Only three patients died, two from a TBI and one from a hemorrhagic shock.

The severity of injuries according to the ISS was significantly higher in males (10.3 vs. 5.8, *p* = 0.009). Moreover, while no statistically significant differences were found with respect to location (inter-urban road: 12.3 ± 9.0; rural area; 6.3 ± 4.5. urban area: 9.1 ± 7.9, *p* = 0.202) or bicycle type (mountain/downhill: 8.3 ± 5.7; road: 14.3 ± 12.3; other: 10.7 ± 9.3, *p* = 0.214), differences were observed with respect to the mechanism of injury and collisions with other agents, which are associated with a significantly higher ISS score (14.7 vs. 8.7, *p* = 0.033). Furthermore, a moderate correlation was found between ISS score and length of hospital stay (r = 0.376). Lastly, patients with a TBI exhibited a significantly higher ISS score (6.4 ± 4.2 vs. 18.1 ± 10.6, *p* < 0.001).

### 3.2. The Helmet as a Key Safety Device

Of the 111 patients for whom helmet use information was available, 71.1% were wearing a helmet at the time of the accident. Helmet use frequencies were similar across the different days of the week (*p* = 0.051), the different months of the year (*p* = 0.690) and the different years analyzed (*p* = 0.655), ranging from a minimum of 42.9% in 2016 to a maximum of 77.8% in 2021.

The relationship between helmet use and the other variables is shown in Table 3, with statistically significant differences found with respect to the location where the accident took place (*p* < 0.001). Specifically, use of a helmet was lower in urban areas than in rural areas (*p* = 0.003) or inter-urban roads (*p* < 0.001).

Patients wearing a helmet presented with a significantly lower neurological AIS score (*p* = 0.031) and were less likely to require transportation by a mobile ICU, with statistically significant differences being found with other types of medical transport (*p* was lower than 0.05 in all cases).

### 3.3. In-Hospital Costs

The total treatment cost for the whole patient cohort was €1,119,433 (€784–€151,505), with the mean per-patient cost standing at €8545 ± €15,298.

The severity of the injuries sustained would seem to have had an impact on the treatment cost (Table 4). A moderate correlation was observed between injury severity and the ISS (r = 0.424), with higher costs being correlated with more severe injuries as evaluated by the ISS score (*p* < 0.001). Costs were, in turn, significantly higher among polytraumatized patients (*p* < 0.001). Lastly, a moderate correlation was found between treatment costs, the number of surgeries performed (r = 0.448) and the neurological AIS score (r = 0.421), and a strong correlation was observed between costs and length of hospital stay (r = 0.921).

## 4. Discussion

The last few years have seen a notable increase in the use of bicycles in Spain, with one-third of the population identifying themselves as regular cyclists [2]. This development emphasizes the need to analyze the various types of cycling accidents that occur, as well as their impact. The present study provides an overview of cycling accidents treated at a Spanish tertiary trauma center by describing the characteristics of cyclists and the accidents they were involved in, as well as the epidemiology and severity of the injuries they sustained, while also exploring factors associated with injury severity, helmet use, and in-hospital costs. The ultimate aim was to generate evidence that may help inform preventive strategies and public health decision-making.

### 4.1. Characteristics of Cycling Accidents

Cycling accident rates have grown moderately but steadily in Spain in recent years: 14.3% in 2017, 16.2% in 2019 and 16.7% in 2022 [2,22]. This coincides with an increase in the number of hospitalizations [16,23]. Contrary to the evidence indicating that cycling accidents are more common in the spring and summer months [24,25,26], seasonality was found not to be a significant factor in our analysis.

The majority of our patients were middle-aged males, which is in line with recent institutional data indicating that 96% of the cyclists involved in accidents are males aged between 45 and 54 years [6]. Although certain discrepancies exist with respect to the age group most at risk, some authors identify cyclists aged between 10 and 19 years and those over 60 as the ones suffering most accidents [27]; it is generally accepted that males are not only more regular bicycle users, but also more prone to cycling accidents. Although the reasons behind these findings remain unclear, they have been attributed to the influence of psychosocial factors such as gender-specific perceptions of danger and females usually choosing quieter and safer roads than males [28,29,30].

According to the information available, urban areas in Spain were the scene of 55% of hospitalizations due to cycling accidents in 2022 [6], which is consistent with the frequency observed in our analysis (56.3%). In our cohort, almost one-third of subjects involved in cycling accidents outside urban areas in our analysis were middle-aged men. It may well be the case that such men were engaging in recreational or sports cycling when they sustained their accident, as, although not specifically collected in our registry, middle-aged men tend to cycle for sports or recreation away from urban environments [23,31,32,33,34]. As regards the mechanism of injury, falls were the most common one in our study, whereas findings in the literature are inconclusive in this regard, with findings varying widely across studies [3,8,35,36].

As mentioned by other authors, the literature on the epidemiology of the musculoskeletal injuries occurring following cycling accidents is not as abundant as might be presumed. Nonetheless, there seems to be a consensus that the most common fractures are those affecting the upper limbs, particularly the clavicle, the forearm and the hand; the face area is also commonly affected [16,23,37,38,39]. Nearly one-third of all fractures in our study occurred in the upper limbs, especially in the clavicle (13.7%).

The widely varying characteristics of the accidents reported in the literature could be due to the specificities of each study in terms of the selected sample, the types of thoroughfares used by cyclists, their reasons for using a bicycle and the lack of a standardized categorization of road types, and the causes leading to a cycling accident [35].

### 4.2. The Importance of the Helmet as a Safety Device

Helmet use among Spanish cyclists is somewhat variable. Between 1993 and 2013, rates stood at 26% [30]. In 2023, 41.4% of cyclists in provincial capitals wore a helmet; that year, 62% of cyclists involved in an accident were wearing a helmet [40]. Helmet use became compulsory in Spain in 1999 on all inter-urban roads, the requirement being extended to persons below the age of 16 in urban areas in 2016 and to persons below 18 in Madrid in 2021 [41,42]. Although evidence on the effectiveness of the legislation on helmet use is inconclusive, there is a general consensus that helmets are not harmful [43,44,45]. Seventy-one percent of subjects in our study were wearing a helmet, and helmet use was considerably more common in areas where it was compulsory.

The overall severity of injuries did not seem to be influenced by whether cyclists were wearing a helmet or otherwise, the evidence in this regard being rather contradictory. While some authors have observed a 28% reduction in the incidence of severe injuries thanks to the use of a helmet [44], as well as lower ISSs in persons wearing a helmet [8], others claim that ISS score differences are not significant [46]. Although the risk homeostasis theory, i.e., the idea that individuals wearing a helmet will take more risks because of a sense of increased protection [47,48,49], is extremely widespread, there are also authors who claim that the evidence to support the said theory is insufficient [50].

Reports in the literature are heterogeneous as regards the protection afforded by the helmet against face injuries. Some claim that the helmet contributes to reducing their occurrence, with an estimated lower incidence ranging between 17 and 47% [43,44,51,52], depending on the exact location of the injury. However, even if the incidence of these injuries is lower, they are not always less severe [53]. It is generally accepted that the protective effect of the helmet on the face is lower than on other parts of the head [43,44,54], which is consistent with our own findings: patients wearing a helmet did not sustain significantly fewer or less severe face fractures, as measured by the AIS for facial injuries.

In contrast, the benefits of helmet use in preventing neurological damage are well documented. It is estimated that wearing a helmet results in a reduction of between 60 and 88% [43] in head injuries and nervous system lesions, constituting the main protection against TBI [46,55], skull fractures [46], brain damage and the need for neurosurgery. While helmet use was not a determining factor in the risk of sustaining a TBI or a skull fracture among our patients, it was a key element in determining their severity; cyclists wearing a helmet exhibited a significantly lower neurological AIS score.

### 4.3. Injury Severity

Over half of our patients presented with injuries of low severity, which coincides with the official data on cycling accidents in Spain [39].

Our results suggest that, of all the variables considered, the only one that proved crucial for determining overall injury severity was mechanism of injury, with collisions resulting in moderately severe injuries and falls resulting in minor injuries. Collisions with other agents tend to result in more severe injuries than falls; clashes with moving vehicles have been identified as a particularly strong determining factor [3,35,36,56]. However, some reports have found collisions with non-motor-driven agents to result in more severe injuries, which may also be explained by the risk homeostasis theory, whereby cyclists may relax their perception of danger in certain areas or at certain times [3]. Given the small sample size of this study, we opted to group all collisions into one single category to ensure uniformity and contribute to a more robust analysis.

In line with previous studies, we chose not to conduct an analysis of the potential association of mortality with the other variables considered, given the low number of deaths recorded [30]. Nonetheless, it is worth mentioning that two of the three deaths that occurred among our patients were caused by a TBI, the most common cause of death among cyclists in Spain and the injury associated with the highest mortality risk among cyclists worldwide [11,16,26].

### 4.4. Economic Impact

The mean per-patient cost stood at €8545. Given the design of the study, only the direct costs associated with the patients’ in-hospital course could be included, so the value obtained is necessarily lower than the actual total cost, which should also comprise the expenses related to the patients’ follow-up and rehabilitation, as well as the potential indirect costs derived from sick leave.

It is difficult to put this figure into perspective, given the scarcity of specific literature on the subject, the economic differences between countries, the varying ways in which healthcare systems are managed, and the effects of inflation over time. That said, the literature reports indicate that the mean direct per-patient cost following a cycling accident is typically lower than the figure obtained in this analysis: €2700 in Finland (2010) [12], €2610 in the Netherlands (2015) [57] and AU$2569 in Australia (2015) [58].

Wearing a helmet did not result in lower costs for our patients. This did not come as a surprise, as no significant differences were observed in the overall severity of injuries between cyclists who wore a helmet and those who did not. Nevertheless, other studies did find helmet use to be associated with a reduction in costs, some of them reporting costs twice as high in cyclists not wearing a helmet [11,59]. Given that TBI is one of the most severe and most feared injuries sustained by cyclists, several studies have focused their attention on the costs associated with such injuries, estimating that they may be twice as high as those corresponding to other lesions. It should be emphasized that the cost of treating a TBI can be up to €62,391 higher among cyclists not wearing a helmet at the time of the accident, which underscores the economic implications of helmet use [11,59]. Costs in our study were significantly higher among patients with a TBI, with the TBI AIS score and the ISS exhibiting a moderate correlation with costs. Moreover, costs were significantly higher in patients with more severe injuries, with polytraumatized patients accounting for a median cost increase of nearly €12,000, in line with previous reports in the Spanish literature [60].

### 4.5. Limitations

Our study has several limitations. The selection of a retrospective design meant that the variables analyzed depended on the information available in the hospital’s records, which limited access to data on the patients’ post-discharge follow-up. In addition, this study is subject to recall bias due to the retrospective nature of the telephone interviews carried out. This limitation is particularly relevant given that data were collected between 1 and 8 years post-injury, which means that some participants were unable to recall all the details of their accident. Also, in the absence of a detailed breakdown of costs, cost calculations were based on an estimation of the main expenses incurred, made available by the hospital management. We believe, however, that these limitations are offset by the fact that this is, to the best of our knowledge, the first-ever description of the management of cycling accidents in a tertiary trauma surgery hospital in Spain over a protracted period of time.

## 5. Conclusions

The cycling accidents analyzed in this study resulted in injuries of minor severity in 64.9% of cases. Injuries were most common in the upper limbs, with three of our patients progressing to death. Helmet use was more widespread in areas where it was compulsory and resulted in less severe neurological lesions, but not a lower incidence of TBIs. The overall severity of injuries sustained following collisions was lower than that of injuries sustained as a result of falls. The mean per-patient cost stood at €8545 and was closely correlated with the patients’ length of hospital stay. The cost for patients with a TBI, as well as for those with a higher TBI AIS score and poor general condition, was higher as they required more costly healthcare resources.

## Figures and Tables

**Table 1:** Characteristics of cycling accidents and the patients’ in-hospital treatment course. The N value indicated for each variable corresponds to the number of patients for whom data were available for the relevant variable. Results are presented as N (%) or as mean ± SD.

Variable	
Location (N = 96)	
Inter-urban road	27 (28.1%)
Rural area	15 (15.6%)
Urban area	54 (56.3%)
Mechanism of injury (N = 129)	
Collision with another agent	21 (16.3%)
Fall	108 (83.7%)
Bicycle type (N = 97)	
Mountain bike/downhill bike	69 (71.1%)
Road bike	21 (21.7%)
Other	7 (7.2%)
Type of transport used (N = 131)	
Ambulance	44 (33.6%)
Helicopter	7 (5.3%)
Mobile ICU	14 (10.7%)
Self-transport	66 (50.4%)
Department patients were admitted to (N = 131)	
Anesthesiology and Resuscitation	4 (3.0%)
Cardiology	1 (0.8%)
Maxillofacial surgery	13 (9.9%)
Thoracic surgery	10 (7.6%)
Intensive medicine	39 (29.8%)
Neurosurgery	7 (5.3%)
Psychiatry	1 (0.8%)
Trauma surgery	56 (42.8%)
Length of hospital stay (days) (N = 131)	7.5 ± 16.3
Number of surgeries (N = 130)	0.7 ± 0.6

**Table 2:** Musculoskeletal injuries and main severity indicators. Results are presented as N (%) or mean ± SD. N = 131 for all the variables.

	Variable	
Fractures		
Lower limbs	Foot	2 (1.1%)
	Ankle	3 (1.6%)
	Tibia	6 (3.3%)
	Femur	4 (2.2%)
	Fibula	2 (1.1%)
	Pelvis	13 (7.1%)
Spine	Vertebral	7 (3.8%)
	Transverse process	3 (1.6%)
	Spinous process	3 (1.6%)
Upper limbs	Clavicle	25 (13.7%)
	Scapula	3 (1.6%)
	Humerus	5 (2.7%)
	Ulna	4 (2.2%)
	Radius	12 (6.6%)
	Hand	8 (4.4%)
Torso	Ribs	23 (12.6%)
	Sternum	1 (0.5%)
Head	Facial	46 (25.1%)
	Skull	13 (7.1%)
Fracture-dislocation		
Lower limbs	Lisfranc	2 (25.0%)
	Ankle	2 (25.0%)
Upper limbs	Ulna	1 (12.5%)
	Galeazzi	3 (37.5%)
Dislocation		
Acromioclavicular		1 (14.3%)
Tarsometatarsal		6 (85.7%)
TBI		39 (30.0%)
AIS		
Lower limbs		0.5 ± 1.0
Spine		0.2 ± 0.7
Upper limbs		0.9 ± 1.0
Abdomen		0.1 ± 0.5
Chest		0.6 ± 1.2
Face		0.5 ± 0.9
Neurological		0.9 ± 1.6
External		0.3 ± 0.4
ISS		9.9 ± 8.6
Severity		
Minor		68 (51.5%)
Moderate		40 (30.3%)
Severe		11 (8.3%)
Very severe		12 (9.1%)
Polytraumatized patients		23 (17.6%)
Mortality		3 (2.3%)

**Table 3:** Relationship between helmet use and accident type, in-hospital course and injury severity parameters. The N value indicated for each variable corresponds to the number of patients with data available for the relevant variable. Results are presented as N (%) or as mean ± SD. Statistically significant comparisons are shown in bold type.

Variable	Helmet Use		*p*-Value
	Yes	No	
Sex (N = 111)			
Males	75 (74.3%)	26 (25.7%)	**0.032**
Females	4 (40.0%)	6 (60.0%)	
Age (N = 111)	43.8 ± 12.9	44.3 ± 16.4	0.873
Location (N = 95)			
Inter-urban road	25 (92.6%)	2 (7.4%)	**<0.001**
Rural area	14 (93.3%)	1 (6.7%)	
Urban area	27 (50.9%)	26 (49.1%)	
Mechanism of injury (N = 110)			
Collision with another agent	9 (47.4%)	10 (52.6%)	**0.024**
Fall	69 (75.8%)	22 (24.2%)	
Bicycle type (N = 97)			
Mountain bike–downhill bike	50 (72.5%)	19 (27.5%)	0.148
Road bike	17 (81.0%)	4 (19.0%)	
Other	3 (42.9%)	4 (57.1%)	
Type of transport (N = 111)			
Ambulance	26 (70.3%)	11 (29.7%)	**0.005**
Helicopter	6 (100%)	-	
Mobile ICU	3 (27.3%)	8 (72.7%)	
Self-transport	44 (77.2%)	13 (22.8%)	
TBI (N = 111)			
Yes	19 (62.5%)	14 (37.5%)	0.065
No	60 (72.6%)	18 (27.4%)	
Skull fractures (N = 111)	5 (56.0%)	4 (44.0%)	0.277
TBI AIS score (N = 111)	0.8 ± 1.6	1.3 ± 1.6	**0.031**
Face fractures (N = 111)	8 (40.0%)	12 (60.0%)	0.344
Face injury AIS score (N = 111)	0.4 ± 0.9	0.5 ± 0.8	0.246
ISS (N = 111)	9.9 ± 7.9	9.8 ± 8.8	0.820
Minor	42 (73.7%)	15 (26.3%)	0.359
Moderate	21 (61.8%)	13 (38.2%)	
Severe	9 (90%)	1 (10.0%)	
Very severe	7 (70.0%)	3 (30.0%)	
Multiple trauma (N = 111)	16 (80.0%)	4 (20.0%)	0.490
Length of hospital stay (N = 111)	8.5 ± 19.8	6.2 ± 8.2	0.896
Number of surgeries (N = 111)	0.7 ± 0.7	0.7 ± 0.6	0.686
Department patients were admitted to (N = 111)			
Anesthesiology and Resuscitation	3 (100%)	-	0.143
Cardiology	-	-	
Maxillofacial surgery	6 (66.7%)	3 (33.3%)	
Thoracic surgery	8 (88.9%)	1 (11.1%)	
Intensive medicine	21 (63.6%)	12 (36.4%)	
Neurosurgery	3 (42.9%)	4 (57.1%)	
Psychiatry	-	1 (100%)	
Trauma surgery	38 (77.6%)	11 (22.4%)	

**Table 4:** Description of the costs corresponding to different degrees of injury severity, the presence of multiple traumas or otherwise, the presence of a TBI or otherwise and the use of a helmet or otherwise. N was equal to 131 for all variables. Statistically significant comparisons are shown in bold type.

Variable	Cost (€)	*p*-Value
Severity		
Low	3.875 (2.905–5.577)	**<0.001**
Moderate	3.984 (2.403–6.536)	
High	10.433 (5.226–19.959)	
Very high	19.756 (11.513–28.162)	
Polytraumatized		
Yes	15.778 (8.137–24.514)	**<0.001**
No	3.949 (2.824–5.912)	
TBI		
Yes	7.341 (3.421–20.292)	**0.002**
No	4.008 (2.862–6.187)	
Helmet use		
Yes	4.430 (2.883–8.916)	0.571
No	3.949 (3.135–5.335)	

## Data Availability

The dataset presented in this article is not readily available due to ethical restrictions.

## Abbreviations

The following abbreviations are used in this manuscript:

AIS	Abbreviated Injury Scale
ISS	Injury Severity Score
RAE-CMBD	Registro de Actividad de Atención Especializada
STROBE	Strengthening of Observational Studies in Epidemiology
TBI	Traumatic Brain Injury
